# Plant flavones enhance antimicrobial activity of respiratory epithelial cell secretions against *Pseudomonas aeruginosa*

**DOI:** 10.1371/journal.pone.0185203

**Published:** 2017-09-20

**Authors:** Benjamin M. Hariri, Derek B. McMahon, Bei Chen, Nithin D. Adappa, James N. Palmer, David W. Kennedy, Robert J. Lee

**Affiliations:** 1 Department of Otorhinolaryngology—Head and Neck Surgery, University of Pennsylvania Perelman School of Medicine, Philadelphia, Pennsylvania, United States of America; 2 Department of Physiology, University of Pennsylvania Perelman School of Medicine, Philadelphia, Pennsylvania, United States of America; Duke University, UNITED STATES

## Abstract

Flavones are a class of natural plant secondary metabolites that have anti-inflammatory and anti-bacterial effects. Some flavones also activate the T2R14 bitter taste receptor, which is expressed in motile cilia of the sinonasal epithelium and activates innate immune nitric oxide (NO) production. Flavones may thus be potential therapeutics for respiratory infections. Our objective was to examine the anti-microbial effects of flavones on the common sinonasal pathogens *Candida albicans*, *Staphylococcus aureus*, and *Pseudomonas aeruginosa*, evaluating both planktonic and biofilm growth. Flavones had only very low-level antibacterial activity alone. They did not reduce biofilm formation, but did reduce production of the important *P*. *aeruginosa* inflammatory mediator and ciliotoxin pyocyanin. However, flavones exhibited synergy against *P*. *aeruginosa* in the presence of antibiotics or recombinant human lysozyme. They also enhanced the efficacy of antimicrobials secreted by cultured and primary human airway cells grown at air-liquid interface. This suggests that flavones may have anti-gram-negative potential as topical therapeutics when combined with antibiotics or in the context of innate antimicrobials secreted by the respiratory or other epithelia. This may have an additive effect when combined with T2R14-activated NO production. Additional studies are necessary to understand which flavone compounds or mixtures are the most efficacious.

## Introduction

Chronic rhinosinusitis (CRS) is a syndrome of chronic inflammation and/or infection of the upper respiratory tract (nose and sinuses, termed the sinonasal cavity), which leads to substantial decreases in patient quality of life, creates >8 billion dollars in direct healthcare costs in the US alone, and can seed lower respiratory infections and exacerbate lung diseases [1–3]. CRS is also an important public health concern, as it accounts for ~20% of antibiotic prescriptions in adults in the US [1, 4–8], making it a significant driver for the emergence of antibiotic resistant organisms [9–15]. Identification of novel compounds with antibacterial and immunomodulatory activity that can be used as topical therapeutics is of paramount importance to combating CRS and other types of respiratory diseases.

Plants produce thousands of polyphenolic flavonoid compounds [16–19], which are of great biomedical interest because they have biological effects on both eukaryotic and prokaryotic cells [17–28]. Flavones are a sub-group of flavonoids that have been demonstrated to have antibacterial, antioxidant, and anti-inflammatory effects in various *in vitro* models [18, 21, 23, 25, 27, 29, 30]. The flavone apigenin is found in bee propolis and chamomile flowers [20]. Apigenin can inhibit the enzyme D-alanine:D-alanine ligase [22], which catalyzes the production of the peptidoglycan precursor D-ala-D-ala, an essential component of the bacterial cell wall. Apigenin may also increase susceptibility of methicillin-resistant *Staphylococcus aureus* (MRSA) to β-lactam antibiotics [23]. Apiginin can inhibit inflammatory protein kinase C (PKC) [31, 32] and nuclear-factor-kappa B (NFκB) [33] signaling in cells *in vitro*. The flavone chrysin is from *Passiflora* and chamomile flowers as well as the *Pleurotus ostreatus* mushroom [18, 21]. Chrysin was previously demonstrated to have both antifungal and antibacterial effects, either alone or in combination with antibiotics [18, 23, 25, 27, 29, 30]. Chrysin may also inhibit inflammatory responses by interfering with cyclooxygenase-2 production of prostaglandins [24, 34]. Wogonin is from *Scutellaria baicalensis*, one of the 50 fundamental herbs of traditional Chinese medicine [35]. Wogonin has antibacterial effects against *Flavobacterium* fish pathogens [36, 37] and also inhibits inflammatory signaling by TNFα [38], PKC [39], and NFκB [40–42] in mammalian cells *in vitro*. Tangeritin is a flavone found in the peels of tangerines and other citrus fruits [18, 21] which may have antibiofilm effects [43].

Moreover, many flavones activate bitter “taste” G-protein-coupled receptor [44–46], known as taste family 2 receptors or T2Rs. T2Rs are now known to be expressed in many tissues outside of the tongue [47, 48], including in motile cilia of the sinonasal cavity, where they modulate mucociliary clearance through activation of nitric oxide (NO) synthase (NOS) [2, 48–53]. We recently demonstrated that several flavones activate the bitter taste receptor isoform T2R14, which is expressed in sinonasal motile cilia [32]. Innate antimicrobial activities of flavones combined with their ability to activate airway T2Rs may increase their potential efficacy as therapeutics to eradicate infections independent of antibiotics. We thus sought to test the effects of flavones on three representative nasal pathogens frequently found in CRS patients [54, 55]: (1) *Candida albicans*, a fungus, (2) *Staphylococcus aureus*, a gram-positive bacteria species, and (3) *Pseudomonas aeruginosa*, a gram-negative bacteria species. Understanding how flavones interact with different types of sinonasal pathogens will begin to elucidate the potential clinical utility of these compounds against bacterial or fungal respiratory infections.

## Results

**Fig 1:**
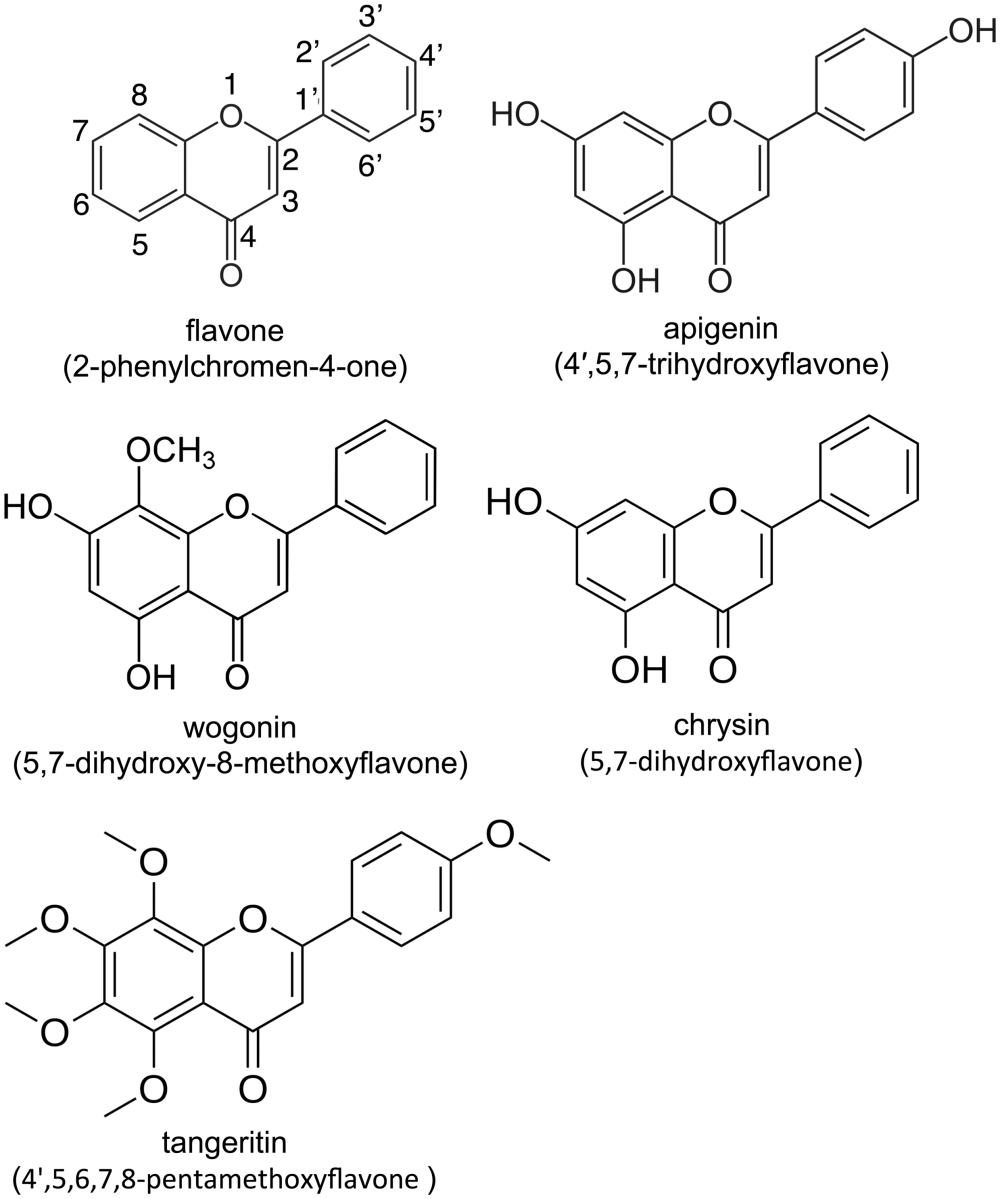
Flavone backbone structure and specific compounds used in this study.

We studied several representative naturally-occurring flavones: agpigenin, chrysin, wogonin, and tangeritin (Fig 1). These flavones have been determined in studies from our own lab [32] and others [44–46] to activate T2Rs and also were determined to have antibacterial effects in other studies (described above). We observed only very subtle effects of individual flavones or a mixture of flavones on planktonic growth of the fungal nasal pathogen *C*. *albicans* (S1 Fig), measured by changes in optical density (OD) at 600 nm (OD_600_). A mixture of apigenin, chrysin, tangeritin, and wogonin (100 μM each) slowed planktonic growth by approximately 50% (*p* <0.01 vs. control). Wogonin was the only individual flavone that significantly reduced OD_600_ after 6 hrs (*p* < 0.05 vs. control) (S1 Fig). The flavone mixture did not significantly enhance the activity of a low concentration of the antifungal antibiotic amphotericin B (0.25 μg/mL). No effects were observed on *C*. *albicans* hyphae growth (S1 Fig), measured using a strain of *C*. *albicans* (HGFP3) expressing GFP under a hyphae-specific promoter [56]. We also found that flavones likewise had only small effects on the growth of two coagulase–negative *Staphylococcus* (CNS) and two methicillin-resistant *S*. *aureus* (MRSA) clinical isolate strains (S2 Fig). Flavones did not appear to enhance the activity of a penicillin/streptomycin mix (S2 Fig). However, we did observe a small increase in growth inhibition of MRSA by a low concentration of methicillin (3 μg/mL; S2 Fig) when combined with this flavone mixture. There was no significant effect of the flavone mixture when combined with a higher concentration (30 μg/mL) methicillin (S2 Fig).

**Fig 2:**
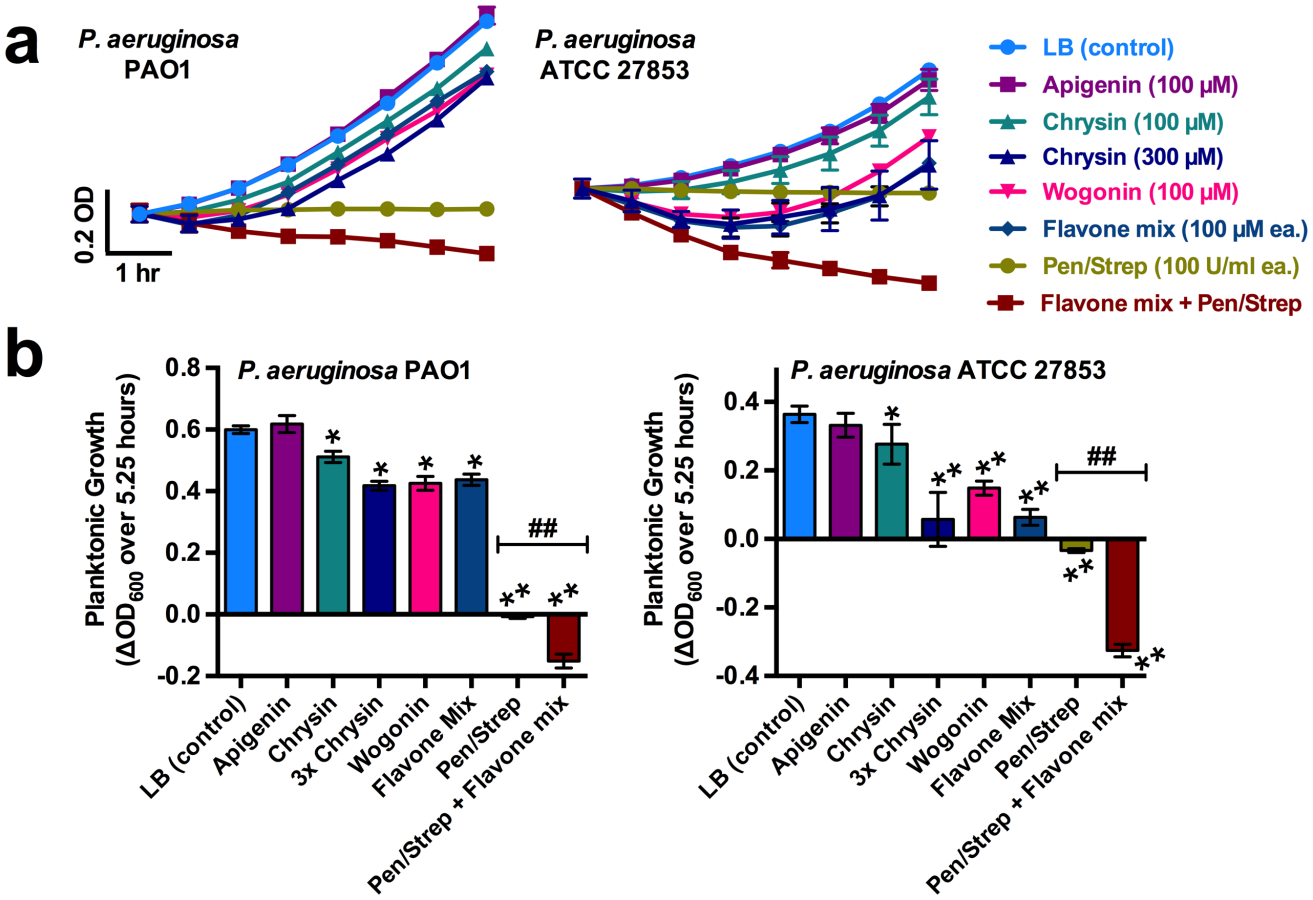
Synergistic anti-bacterial effects of flavones in combination with antibiotics. (A) Planktonic growth traces (OD_600_) of 2 strains of *P*. *aeruginosa* (PAO1 and ATCC 27853) under the indicated conditions. Note reduction of OD_600_ in the presence of penicillin/streptomycin plus flavone mixture (apigenin, chrysin, wogonin; 100 μM each). (B) Bar graphs showing ΔOD_600_ over 5.25 hrs from *A* (n = 4 experiments for each condition). Asterisks denote significance vs. control (LB only; one-way ANOVA, Dunnett’s post-test; * = *p* <0.05, ** = *p* <0.01); ## indicates *p* <0.01 between bracketed bars (one-way ANOVA, Bonferonni post-test).

In contrast with the more subtle effects observed with *C*. *albicans* and *Staphylococcus*, we saw more marked effects with *P*. *aeruginosa*, an important opportunistic gram-negative airway pathogen [55]. A flavone mixture (apigenin, chrysin, and wogonin; 100 μM each) and some individual flavones significantly reduced planktonic growth, but more interestingly also caused a substantial reduction of culture optical density at 600 nm (OD_600_) when combined with penicillin/streptomycin (Fig 2A and 2B) in wild-type (Wt) *P*. *aeruginosa* strains PAO1 and ATCC 27853. This suggested that flavones may enhance lysis of *P*. *aeruginosa* under certain conditions, possibly by destabilizing cell wall components such as D-ala-D-ala, as previously shown with apigenin [22]. We performed planktonic growth/lysis assays in media of varying ionic strength (100% LB or 25% LB, diluted from 100% with sterile DI-water), and found that lysis of bacteria (evidenced by reduction in OD_600_ from the starting OD of 0.1) in the presence of gentamicin ± flavones was enhanced by low ionic strength (S3 Fig). In the absence of flavones, a drop in OD_600_ was not observed over 6 hrs (S3 Fig), but lysis was observed when gentamicin was combined with flavones. Reduced ionic strength caused a lower OD_600_ (i.e. a greater decrease from the starting OD_600_ of 0.1) at 30 min (S3 Fig).

We also tested flavones against *P*. *aeruginosa* biofilm formation using a 96-well plate-based crystal violet assay, but no effects on biofilms were observed (S4 Fig). However, individual flavones, as well as a flavone mixture, significantly decreased production of the ciliotoxin and inflammatory stimulant pyocyanin [57–61] at effective concentrations (ECs) of 1–10 μM for individual flavones and 0.1 μM for the mixture in planktonic PAO1 and ATCC 27853 cultures (S4 Fig). Despite a lack of effect on *P*. *aeruginosa* biofilms, the data above suggest that flavones have some small but statistically significant effects on bacterial growth alone. More intriguingly, these compounds may significantly enhance the efficacy of antibiotics under certain conditions, potentially by contributing to a disruption of cell wall integrity.

**Fig 3:**
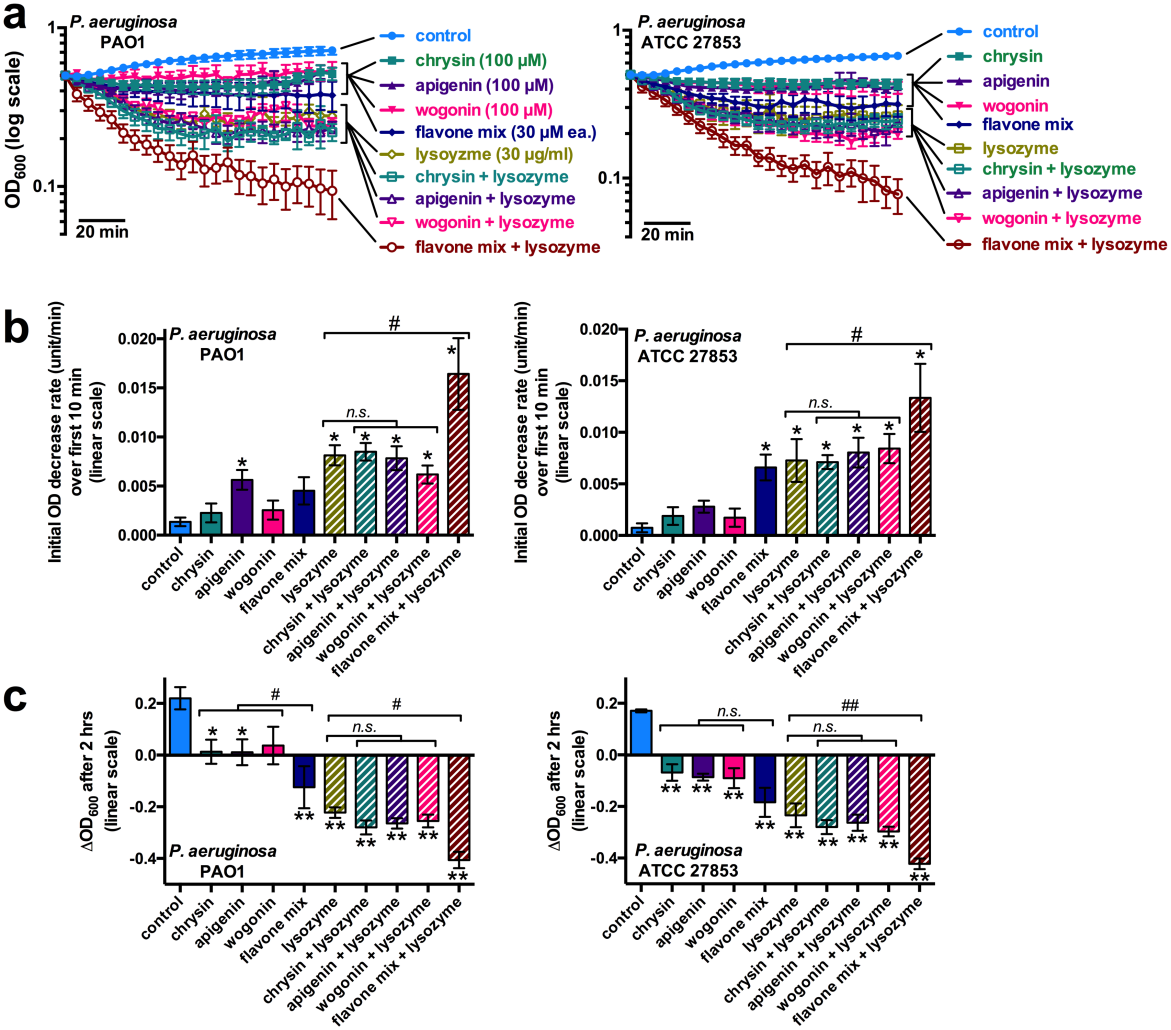
Synergistic anti-bacterial effects of flavones in combination with the airway antimicrobial protein lysozyme. (A) Traces of planktonic growth of *P*. *aeruginosa* in the presence of flavones ± lysozyme. Note the greatest OD_600_ decrease (bacterial lysis) occurred with lysozyme and flavone mix combined. (B) Bar graphs of the initial OD decrease rate (OD_600_ units/min) from *A* (n = 3–6 experiments for each condition). (C) Bar graphs showing ΔOD_600_ after 2 hours from *A*. For *B* and *C*, asterisks denote significance vs. control (LB only) by one-way ANOVA, Dunnett’s post-test (* = *p* <0.05, ** = *p* <0.01); # and ## indicates *p* <0.05 and 0.01, respectively, between bracketed bars (one-way ANOVA, Bonferonni post-test).

Because many innate defense proteins secreted by the airway epithelium act in part through bacterial lysis or permeabilization (e.g. lysozyme [62–67] and β-defensins [68–71]), we sought to test whether flavones may enhance the efficacy of airway antimicrobials. We tested the effects of flavones combined with the important airway antimicrobial lysozyme, which is secreted by airway submucosal gland serous cells [72–74] and catalyzes the hydrolysis of 1,4-beta-linkages between N-acetyl-D-glucosamine and N-acetylmuramic acid residues of peptidoglycan in the bacterial cell wall [75, 76]. We focused on *P*. *aeruginosa*, as it appeared to be the most sensitive to flavone effects among the pathogens tested here. While lysozyme is more effective against gram-positive bacteria, it does have effects against gram-negative bacteria. In the presence of EDTA to help disrupt the outer lipopolysaccharide-containing gram-negative layer, rapid lysis is observed with lysozyme treatment at low ionic strength [76, 77]. Rapid lysis of *P*. *aeruginosa* (decrease in OD_600_) was observed in the presence of recombinant human lysozyme ± flavones (Fig 3A–3C). While individual flavones (apigenin, chrysin, and wogonin) caused minimal lysis under these conditions and did not enhance lysozyme-mediated lysis, a mixture of these flavones (100 μM each) promoted lysis approximately 50% as well as lysozyme after 2 hrs. (Fig 3C). Moreover, the flavone mixture significantly enhanced lysozyme-mediated *P*. *aeruginosa* lysis (Fig 3B and 3C). Data are presented as both initial rate of lysis (Fig 3B) as well as the change in OD_600_ after 2 hrs from the starting OD of 0.5 (Fig 3C).

**Fig 4:**
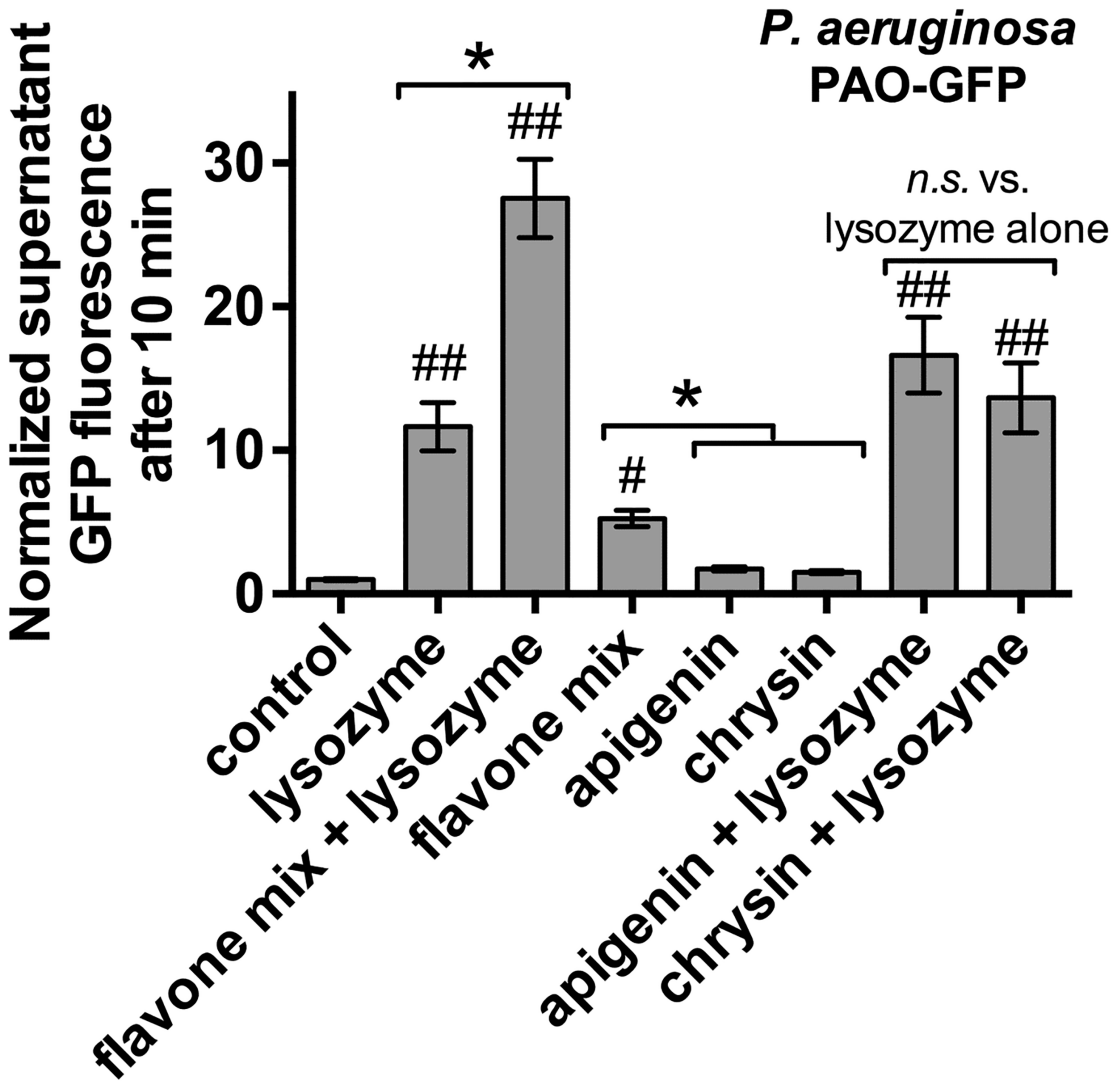
Confirmation of bacterial lysis by measurement of GFP release from PAO-GFP. Bar graph showing normalized fluorescence (control = 1) from PAO-GFP cultures incubated in lysozyme lysis buffer with addition of lysozyme and/or flavones as indicated and described in the text. Flavone mix contained 100 μM each apigenin, chrysin, and wogonin. Apigenin and chrysin were used alone at 300 μM to compare an equal number of moles of flavone molecules. Synergistic effects of the flavone mixture were observed both alone and combined with lysozyme. Significance determined by one-way ANOVA with Bonferonni post-test; # and ## indicate *p* <0.05 and *p* <0.01, respectively, compared with control; * indicates *p* <0.05 between bracketed groups.

To confirm that the decrease in OD_600_ observed reflected bacterial lysis, we quantified GFP release from a strain of *P*. *aeruginosa* PAO1 that expresses soluble GFP, PAO-GFP. After exposure to lysozyme ± flavones for 10 min, cells were centrifuged and supernatant was collected for measurement of fluorescence. Lysozyme significantly increased supernatant fluorescence over control conditions (lysis buffer alone), which was enhanced by addition of the flavone mix (100 μM each apigenin, chrysin, and wogonin) (Fig 4). The flavone mix by itself caused a significant increase in GFP release that was not observed by an equal amount of a single flavone (300 μM apigenin or 300 μM chrysin) (Fig 4). Neither 300 μM apigenin nor 300 μM chrysin significantly increased lysozyme-mediated lysis (Fig 4), supporting that there is a synergistic effect of mixing flavone compounds.

**Fig 5:**
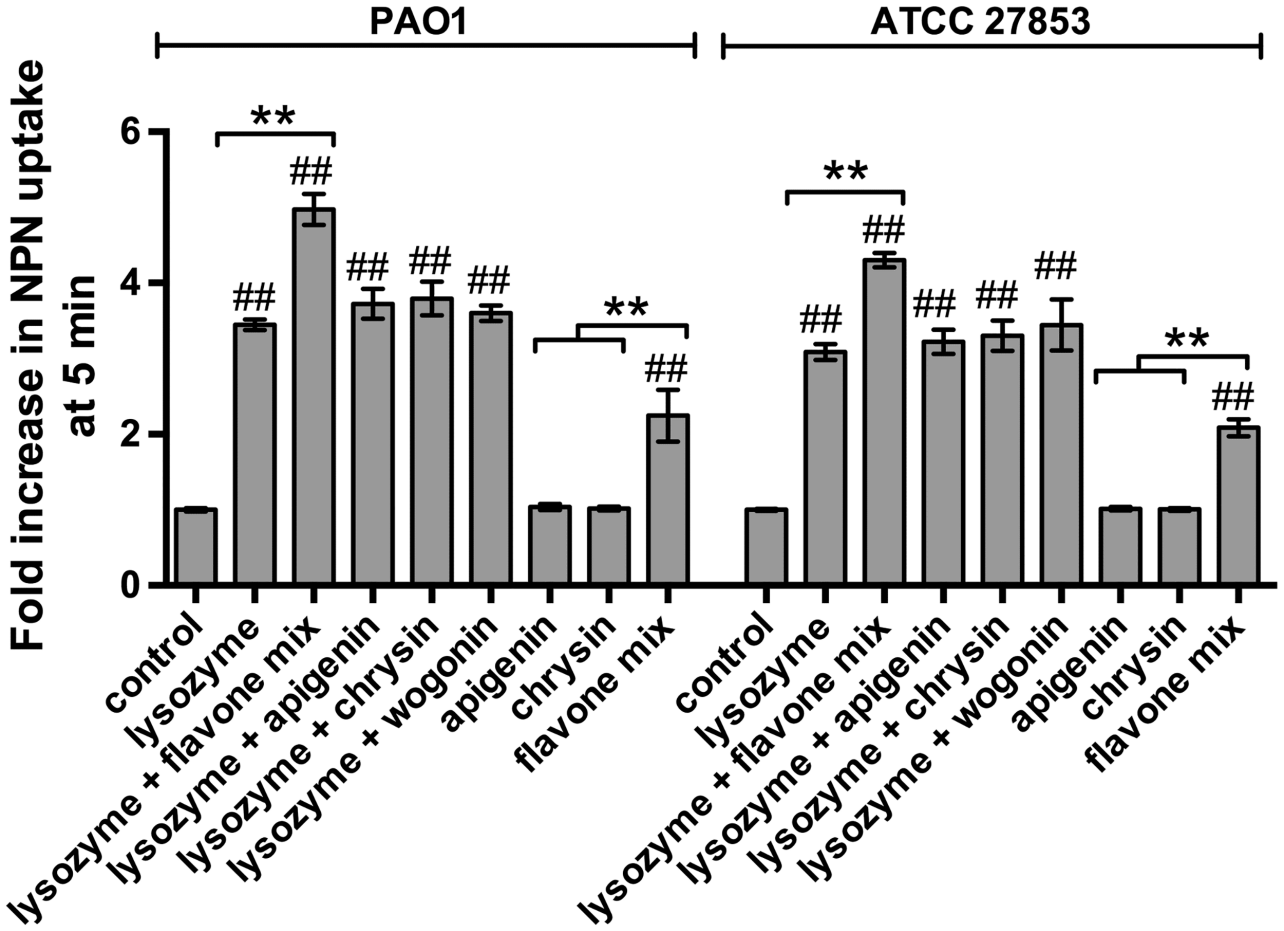
Confirmation of bacterial cell wall damage by NPN uptake. Bacteria were incubated for 5 min with lysozyme ± flavones as indicated and described in the text, followed by measurement of NPN fluorescence, reflecting uptake of NPN into the bacterial phospholipid membrane. Data are expressed as fold increase in NPN fluorescence. Raw fluorescence values are in S1 File. Significance determined by one-way ANOVA with Bonferonni post-test; # and ## indicate *p* <0.05 and *p* <0.01, respectively, compared with control; ** indicates *p* <0.01 between bracketed groups.

An enhancement of cell membrane damage by flavones was also confirmed by staining with 1-N-phenylnapthylamine (NPN). NPN is a hydrophobic fluorophore that undergoes a quantum yield increase upon transition from a hydrophilic aqueous environment to a hydrophobic phospholipid environment [78], and NPN uptake is frequently used to measure gram-negative outer membrane permeabilization [78–82]. A flavone mix enhanced NPN uptake alone or in combination with lysozyme greater than an equal amount of individual flavones, as found above (Fig 5).

**Fig 6:**
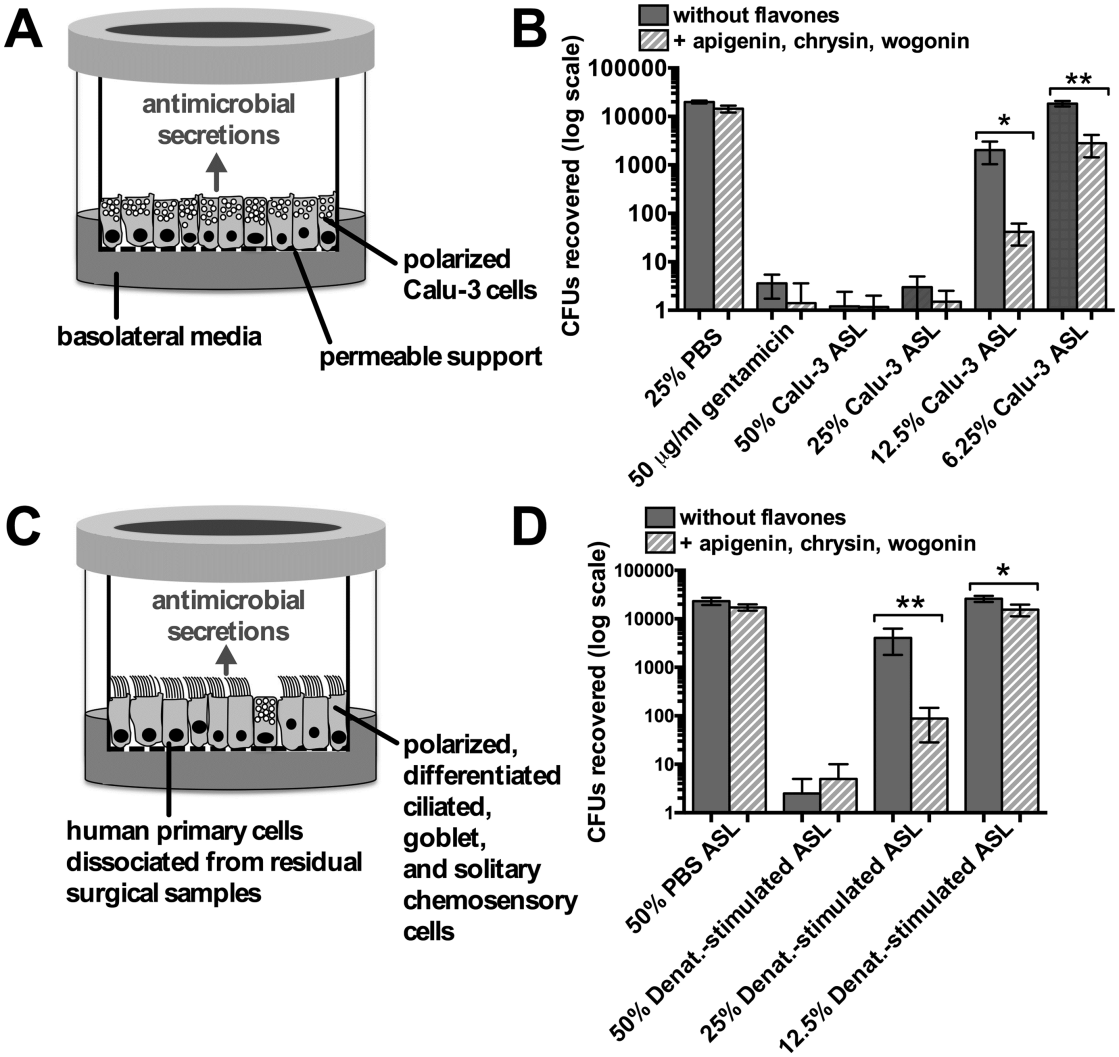
Antimicrobial effects of Calu-3 and primary cell airway surface liquid (ASL) are enhanced by flavones. (A) Calu-3 air-liquid interface cultures (ALIs) recapitulate a polarized secretory epithelium with polarized secretion of antimicrobial peptides and mucus, similar in composition to that of airway submucosal exocrine gland serous acinar cells [65, 72, 89]. (B) Bar graph shows number of colony forming units (CFUs) recovered from bacteria mixed with dilutions of Calu-3 ASL washings. As negative control, bacteria were incubated with 25% PBS (first column) not in contact with Calu-3 cells; 50 μg/mL gentamicin in 25% PBS was used as positive control. Antimicrobial activity was enhanced at lower dilutions of Calu-3 ASL (12.5% and 6.25%) in the presence of the flavone mix (50 μM each apigenin, chrysin, and wogonin). (C) Primary sinonasal epithelial cultures recapitulate the surface airway epithelium, with differentiated ciliated, goblet, and solitary chemosensory cells, likewise with polarized secretion of antimicrobial peptides and mucus. (D) Bar graph shows CFUs when *P*. *aeruginosa* were mixed with ASL washings from primary sinonasal ALI cultures stimulated with denatonium benzoate (10 mM). Asterisks denote significance determined by one-way ANOVA, Bonferroni post-test of paired columns (each condition ± flavone; * *p* <0.05 and ** *p* <0.01).

To determine if the effects observed above with recombinant lysozyme translated to enhancement of physiological antimicrobials secreted by airway epithelial cells, we tested the ability of flavones to enhance the antimicrobial activity of Calu-3 cell secretions. Calu-3 cells are a model of airway secretory cells [72], resembling some aspects of both submucosal gland serous cells (e.g. secretion of lysozyme [83] and β-defensins [84]) as well as surface epithelial goblet cells (e.g. secretion of Muc5AC [85, 86]). We carried out an antimicrobial assay using apical airway surface liquid (ASL) washings from Calu-3 air-liquid interface (ALI) cultures (ALIs; Fig 6A). We found that the collected Calu-3 washings were potently antibacterial, as they caused a >3-log reduction in the number of *P*. *aeruginosa* CFUs recovered even at a 25% dilution (Fig 6B). The antimicrobial effect was partially lost at further dilution (12.5%) and fully lost at 6.25% dilution, but the activity of these low dilutions was significantly enhanced by the presence of a flavone mixture (Fig 6B).

We also tested the ability of flavones to enhance the activity of ASL washings from primary sinonasal ALIs (Fig 6C), which differentiate into ciliated and goblet cells, mimicking the *in vivo* epithelium and secreting a similar array of antimicrobial peptides [50, 53, 87, 88]. ALI cultures also contain solitary chemosensory cells, which express T2R bitter taste receptors and regulate secretion of antimicrobial peptides from surrounding cells [50]. Primary sinonasal ALIs were stimulated apically with denatonium benzoate, a bitter compound that activates the T2Rs in solitary chemosensory cells (T2Rs 10, 30, and/or 46) and stimulates rapid secretion of stored antimicrobial peptides, including β-defensins 1 and 2 [50]. β-defensins are small cationic proteins that permeabilize bacterial cell membranes. Flavones had no significant effect on CFUs recovered when mixed with control (un-stimulated, PBS-only-treated) ASL (Fig 6D), but potentiated the antibacterial effects of denatonium-stimulated ASL when diluted to 25% and 12.5% (Fig 6D).

## Discussion

**Fig 7:**
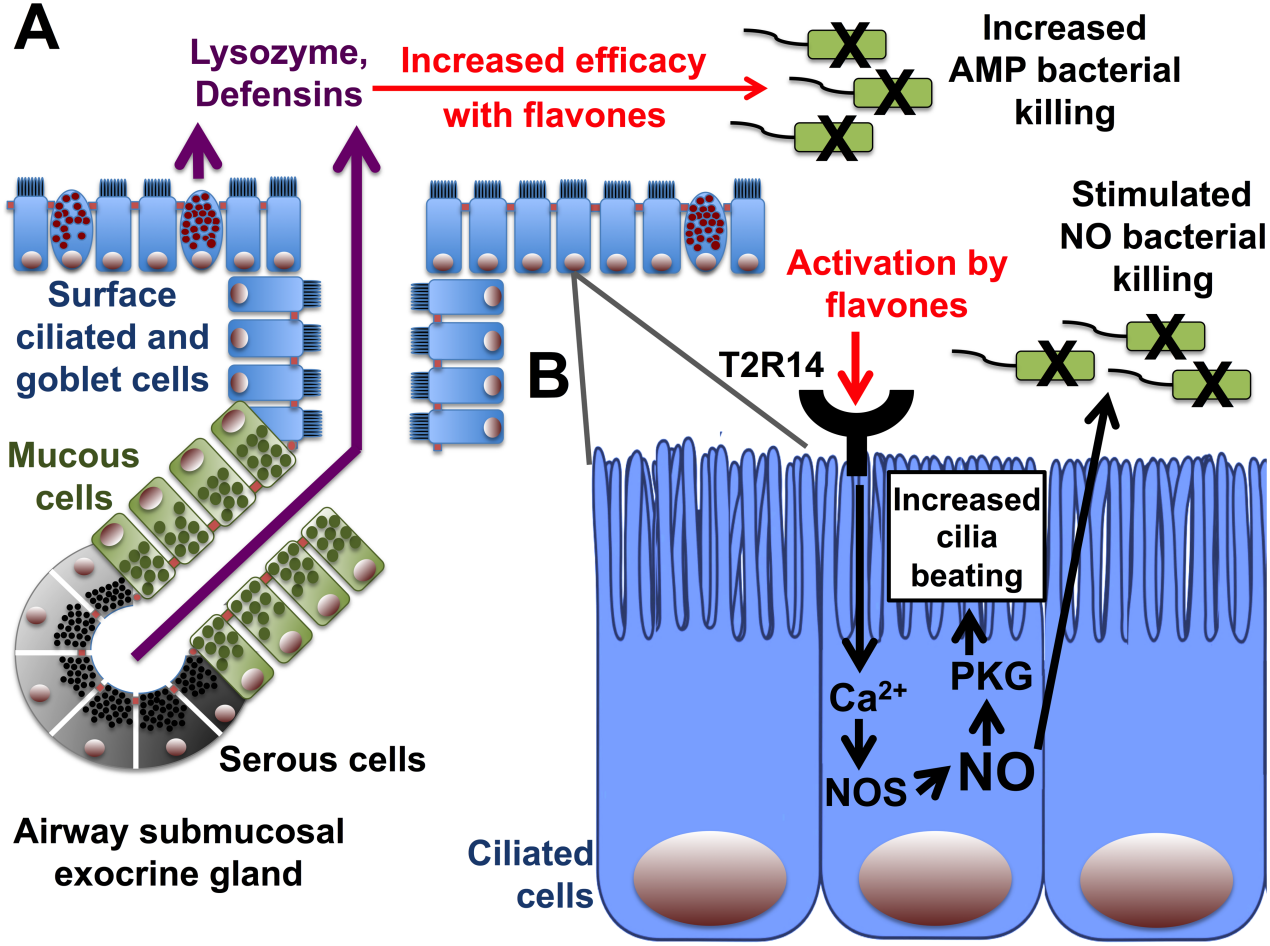
Multiple mechanisms of modulation of respiratory epithelial innate immunity by flavones. (A) Lysozyme is primarily secreted by serous cells of airway submucosal exocrine glands [72]. Defensins are secreted by surface epithelial cells as well as glands. Here, we show that flavones increase the efficacy of these and possibly other secreted antimicrobial peptides (AMPs). (B) We showed previously that flavones also activate the bitter taste receptor T2R14, expressed in both sinonasal [32] and bronchial cilia [99]. T2R14 activation in sinonasal cilia increases nitric oxide synthase (NOS)-mediated production of NO, which increases ciliary beating through protein kinase G (PKG) to promote bacterial clearance and directly diffuses into the airway surface liquid to kill bacteria [53].

Here, we found specifically that a mixture of flavones can enhance the bacteriolytic activity of recombinant human lysozyme against a common gram-negative opportunistic pathogen, *P*. *aeruginosa*, and flavones can enhance the bactericidal activity of endogenous human respiratory cell secretions, of which lysozyme is a major component [65, 83, 89, 90]. Together with our previous study [32], our data suggest flavone compounds might modulate respiratory innate immunity through multiple mechanisms (Fig 7). These compounds may have some clinical utility to enhance antibiotic efficacy or enhance endogenous innate immunity.

To our knowledge, such synergistic activities of a mixture of different but highly similar-structured flavonoids have not been previously reported. While synergistic activities with antibiotics have been reported, synergy with endogenous airway antimicrobials is also a novel finding. While our data suggest that flavones have only very low-level antibacterial activity on their own, they may have a substantial ability to enhance the efficacy of innate antimicrobials secreted by human airway cells. Coupled with their previously demonstrated anti-inflammatory activities [18, 21, 91, 92] and activation of T2R-mediated innate immune responses [32], flavones are an attractive novel class of compounds to investigate as potential topical therapeutics for respiratory infections. The potential clinical utility of these compounds is most strongly supported by their ability to enhance bactericidal efficacy of secretions from human airway cells independent of their NO-generating function on T2R receptors [32], as the flavones were mixed with airway cell secretions after removal from the epithelial cells. Future high-throughput screening of flavone compounds using *in vitro* assays as described here may identify the most efficacious single compounds or mixtures to activate these effects, suggesting which compounds to prioritize for future *in vivo* testing.

## Materials and methods

### Reagents

Unless indicated, all reagents, solutions, and protocols used were as previously described [49, 50, 53, 87, 88]. Stock solutions of flavones (Cayman Chemical, Ann Arbor, MI) were made at 100 or 120 mM in DMSO (≥1000x). Final DMSO concentrations were always ≤0.3%, which had no effect alone on bacterial growth kinetics in any assay tested. Reagents not specifically listed below were obtained from Sigma-Aldrich (St. Louis, MO).

### Bacterial and fungal culture and antibacterial assays

*Pseudomonas aeruginosa* strains PAO1 (HER-1018; ATCC BAA-47) and ATCC 27853 (Boston 41501) were from American Type Culture Collection (Manassas, VA) cultured in LB medium (Gibco/Thermo Scientific). Methicillin-resistant *Staphylococcus aureus* (MRSA) strain M2 [93] and clinical isolates of MRSA and coagulase-negative *Staphylococcus* (isolated by the Philadelphia VA Medical Center Microbiology Laboratory) were grown in tryptic soy broth (TSB; Gibco/Thermo Scientific). *Candida albicans* strain HGFP3[56] (kindly provided by Drs. S. Sundstrom, N. Kavanaugh, and K. Ribbeck) was grown in YPD broth (Gibco/Thermo Scientific). Antibiotics (gentamicin, methicillin, and penicillin/streptomycin mixture) were obtained from Sigma. PAO-GFP, a gift from Dr. N. Cohen (University of Pennsylvania), expresses soluble GFP on a gentamicin-resistant plasmid and was grown in media containing 50 μg/mL gentamicin to maintain selection.

For planktonic growth assays, an overnight log-phase culture was diluted to a density of 0.1, with 10 mL total volume per sample. Cultures were grown in 15 mL tubes at 37°C with shaking (180 RPM); 1 mL of solution was removed at each time point (up to 10 time points) and assayed for optical density (OD) at 600 nm in a spectrophotometer. Biofilm assays were carried out in 96-well plates as previously described [28, 94]. After incubation for 48 hrs, microtiter plates were washed with distilled water, followed by staining with 1% crystal violet for approximately 30 min. After a second washing, biofilm mass and crystal violet were solubilized by incubation in 30% acetic acid for 30 min with shaking, and read on a plate reader at 590 nm. Pyocyanin extraction was carried out as previously described [95]. Briefly, 8 mL of supernatant from an overnight culture (grown in cation-adjusted Mueller-Hinton broth, normalized to OD_600_ = 1) was mixed with 3 mL of chloroform. After vortexing and centrifugation, pyocyanin was extracted from the resulting organic chloroform phase with 1 mL of 0.2 N HCl, with absorbance of the acidified pyocyanin read at 520 nm in a plate reader (Spark 10M, Tecan, Männedorf, Switzerland). All values were blanked to LB that had undergone the same extraction procedure. Lysozyme lysis assays were conducted essentially as described [76, 77, 96]. Bacteria were washed and resuspended (OD 0.5) in 20 mM Tris-HCl, 1 mM EDTA, pH 8.0 with recombinant human lysozyme ± flavones before monitoring OD_600_ between incubations at 37°C. For GFP release assays, lysozyme lysis experiments were carried out as above in 1 mL volume; after 10 min, samples were centrifuged to pellet bacteria (10,000 *g* for 15 min) and supernatant GFP fluorescence was measured on a Tecan Spark 10M plate reader (485 excitation, 535 emission). Background was estimated by measuring lysis buffer alone and was subtracted from each experimental value before normalization to control conditions (supernatant from cells in lysis buffer alone, no lysozyme).

Bacterial NPN fluorescence assay was modified from previous descriptions [79–82]. *Pseudomonas* were grown to an OD_600_ of 0.5 in LB, centrifuged, and resuspended at half volume of 10 mM HEPES, 5 mM glucose, 0.1 mM EDTA, pH 8. Bacteria were then aliquoted and mixed with an equal volume of diluted airway surface liquid secretions or antibiotics, and then pipetted into a plate reader containing an equal volume of 25% PBS containing 20 μM NPN (final NPN 10 μM, final OD_600_ 0.25). Samples were then incubated for 10 min and read on a Tecan 10M plate reader at 350 nm excitation and 450 nm emission. Emission wavelength was chosen to minimize any endogenous fluorescence of flavones used. Samples were read in triplicate, with averages of at least 3 independent experiments reported. At these wavelengths, flavones at concentrations used exhibited no significant fluorescence compared with buffer alone.

CFU antimicrobial assays with ASL washings were carried out similarly to a previously published protocol [50, 90] and modified based on our own antimicrobial ASL protocols used in our lab [50]. Cultures were washed copiously with PBS and transferred to antibiotic-free MEME for 48 hrs. before use. Calu-3 cell secretions were collected from 3 week old ALIs stimulated basolaterally with 100 μM isoproterenol for 72 hours, followed by washing of the apical surface with 30 μL 25% PBS. While washing a 1.1 cm^2^ ALIs with 30 μL significantly dilutes the ASL fluid (~1 μL per cm^2^ of surface area [97]), washings retained antibacterial activity and were thus sufficient to be used for this assay. ASL washings (30 μL per culture) were pooled and mixed with bacteria resuspended in 25% PBS, adjusted to 0.1 OD, then diluted 1:1000 in 25% PBS). Bacteria/ASL mixture was divided and flavone mixture (1000x stock in DMSO) was added. Bacteria and ASL mixture was incubated statically in a 96-well plate at 37°C for 2 hrs, followed by 4 serial 10-fold dilutions and spot plating onto LB plates. After overnight incubation at 37°C, CFUs were manually counted.

For primary sinonasal cultures, we carried out an antimicrobial assay as previously described [50]. ASL cultures (0.33 cm^2^ surface area) were transferred to antibiotic free medium for 48 hrs. and washed copiously on the day of the experiment to remove already-secreted antimicrobials. ALIs were then treated on the apical side with 30 μL of 25% PBS ± 10 mM denatonium benzoate and incubated for 30 min at 37°C. ASL was then collected from 3–4 cultures from the same patient and pooled, followed by further dilution with 25% PBS and/or mixing 1:1 with bacteria diluted in 25% PBS (0.1 OD overnight culture diluted 1:1000 in 25% PBS ± flavones). Bacteria and ASL were incubated for 2 hrs at 37°C followed by dilution and spot plating as described above for Calu-3 cells.

### Generation of Calu-3 and primary sinonasal air-liquid interface (ALI) cultures

Calu-3 bronchial epithelial cells were obtained from ATCC and cultured in T75 flasks in minimal essential medium (MEM) with Earl’s salts and 1 mM L-glutamine, 10% fetal bovine serum, and 1% cell culture penicillin/streptomycin mix. Cells were lifted with 0.25% trypsin and plated on 1.1 cm^2^ cell culture inserts (Greiner BioOne Thincerts, transparent, 0.4 μm pore size). Cells were grown to confluence for 5 days, followed by apical exposure to air and subsequent 3–4 weeks for full differentiation/polarization before use. Only ALIs with transepithelial resistances (TEERs) of 250–300 Ω·cm^2^ were used.

For primary cells, all experimental protocols were carried out in accordance with the University of Pennsylvania School of Medicine guidelines regarding use of residual clinical material in research, U.S. Department of Health and Human Services code of federal regulation Title 45 CFR 46.116, and the Delcaration of Helsinki. Patients undergoing sinonasal surgery for either sinonasal disease (e.g. chronic rhinosinusitis) or other procedures (e.g. transnasal approaches to the skull base) were recruited from the Department of Otorhinolaryngology at the University of Pennsylvania with full IRB approval (#800614) and written informed consent was obtained for all participating. Inclusion criteria included patients over 18 years of age who were undergoing medically necessary sinonasal surgery. Exclusion criteria included a history of systemic diseases (e.g. granulomatosis with polyangiitis, cystic fibrosis), immunodeficiencies (e.g., common variable immune deficiency), or use of antibiotics, oral corticosteroids, or anti-biologics (e.g. Xolair) within one month of surgery. Human sinonasal epithelial cells were enzymatically dissociated and grown to confluence in proliferation medium (DMEM/Ham’s F-12 plus BEBM; Clonetics, Cambrex, East Rutherford, NJ) for 7 days as previously described [53, 98]. Confluent cells were dissociated and seeded on porous polyester membranes coated with BSA, type I bovine collagen, and fibronectin in Corning Transwell cell culture inserts in LHC basal medium (Invitrogen). Culture medium was removed from the upper compartment and basolateral media was changed to differentiation medium (1:1 DMEM:BEBM) containing hEGF (0.5 ng/mL), epinephrine (5 ng/mL), BPE (0.13 mg/mL), hydrocortisone (0.5 ng/mL), insulin (5 ng/mL), triiodothyronine (6.5 ng/mL), and transferrin (0.5 ng/mL), supplemented with 100 U/mL penicillin, 100 g/mL streptomycin, 0.1 nM retinoic acid, and NuSerum (BD Biosciences, San Jose, CA) as previously described [53, 98].

### Data analysis and statistics

One-way analysis of variance (ANOVA) was performed in GraphPad Prism with appropriate post-tests as indicated; *p* <0.05 was considered statistically significant. For comparisons of all samples within a data set, Tukey-Kramer post-test was used. For preselected pair-wise comparisons, Bonferroni post-test was performed. For comparisons to a single control group, one-way ANOVA with Dunnett’s post-test was used. All other data analysis was performed in Microsoft Excel. For all figures, one asterisk or pound sign (* or #) indicates *p* <0.05 and two asterisks or pound signs (** or ##) indicates *p* <0.01 respectively; “*n*.*s*.” indicates no statistical significance. All data are presented as mean ± SEM.

## Supporting information

S1 Fig Flavones have minimal effects on Candida abicans growth. (PDF)

S2 Fig Effects of flavones on planktonic *Staphylococcus* growth. (PDF)

S3 Fig Effects of ionic strength on flavone + antibiotic cell lysis. (PDF)

S4 Fig Flavones do not affect *P*.*aeruginosa* biofilm formation but do reduce pyocyanin production. (PDF)

S1 File Data values from the main text. (PDF)
