# Supplementary material for: Plant flavones enhance antimicrobial activity of respiratory epithelial cell secretions against Pseudomonas aeruginosa
Source: PLoS One. 2017 Sep 20;12(9):e0185203. doi: 10.1371/journal.pone.0185203 (PMC5607194; doi:10.1371/journal.pone.0185203)
Supplement: S1 Fig — (PDF) [file pone.0185203.s001.pdf]

Supporting Information

Hariri, *et al.* "Plant Flavones Enhance Antimicrobial Activity of Respiratory Epithelial Cell Secretions Against *Pseudomonas aeruginosa*"

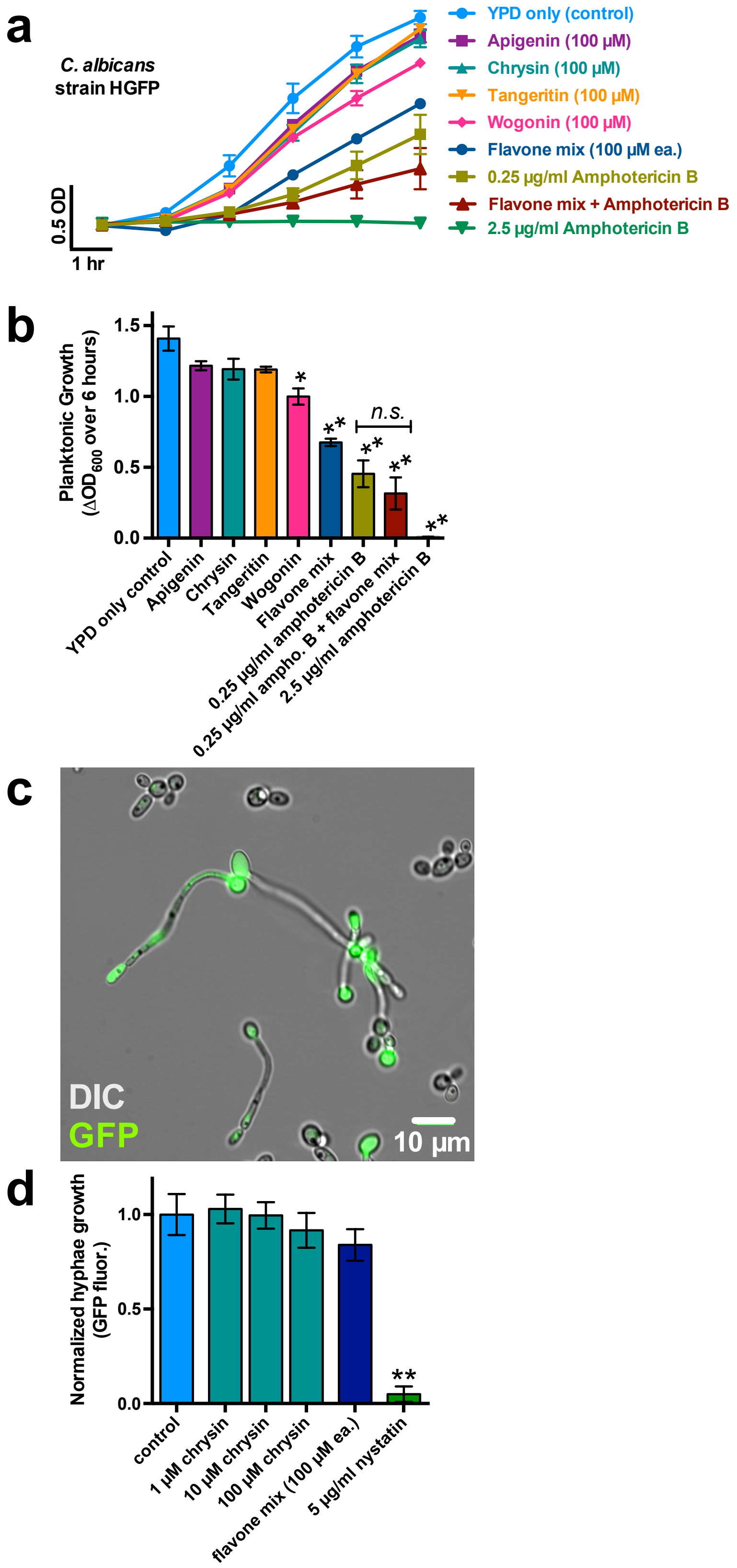

S1 Fig. Bitter flavones have minimal effects on Candida abicans planktonic or hyphae growth. (A) Planktonic growth of C. albicans in the presence of flavones individually, or as a mixture in the presence or absence of amphotericin B; n = 3-8 experiments for each condition. (B) Bar graph showing growth after 6 hours. Wogonin was the only flavone that individually reduced the change in OD600 (1.0 ± 0.06 vs. 1.4 ± 0.09 with YPD only control; p <0.05). A flavone mix (100 µM each apigenin, chrysin, tangeritin, and wogonin) also reduced planktonic growth (0.98 ± 0.03; *p* <0.01 vs control), as did both concentrations of amphotericin B. The planktonic growth with 0.25 µg/ml amphotericin B (0.45 ± 0.09) was not significantly different in the presence of the flavone mix (0.31 ± 0.11). (C) Image of HGFP3 C. albicans, showing high GFP expression in hyphae but not planktonic cells. (D) Hyphae growth was measured in a fluorescence plate reader, and fluorescence was not significantly altered by three concentrations of chrysin or the same flavone mix used above. Fluorescence was reduced by 5 µg/ml nystatin. For all graphs, * = *p* <0.05, ** = *p* <0.01.
