# Supplementary material for: Plant flavones enhance antimicrobial activity of respiratory epithelial cell secretions against Pseudomonas aeruginosa
Source: PLoS One. 2017 Sep 20;12(9):e0185203. doi: 10.1371/journal.pone.0185203 (PMC5607194; doi:10.1371/journal.pone.0185203)
Supplement: S2 Fig — (PDF) [file pone.0185203.s002.pdf]

Supporting Information

Hariri, *et al.* "Plant Flavones Enhance Antimicrobial Activity of Respiratory Epithelial Cell Secretions Against *Pseudomonas aeruginosa*"

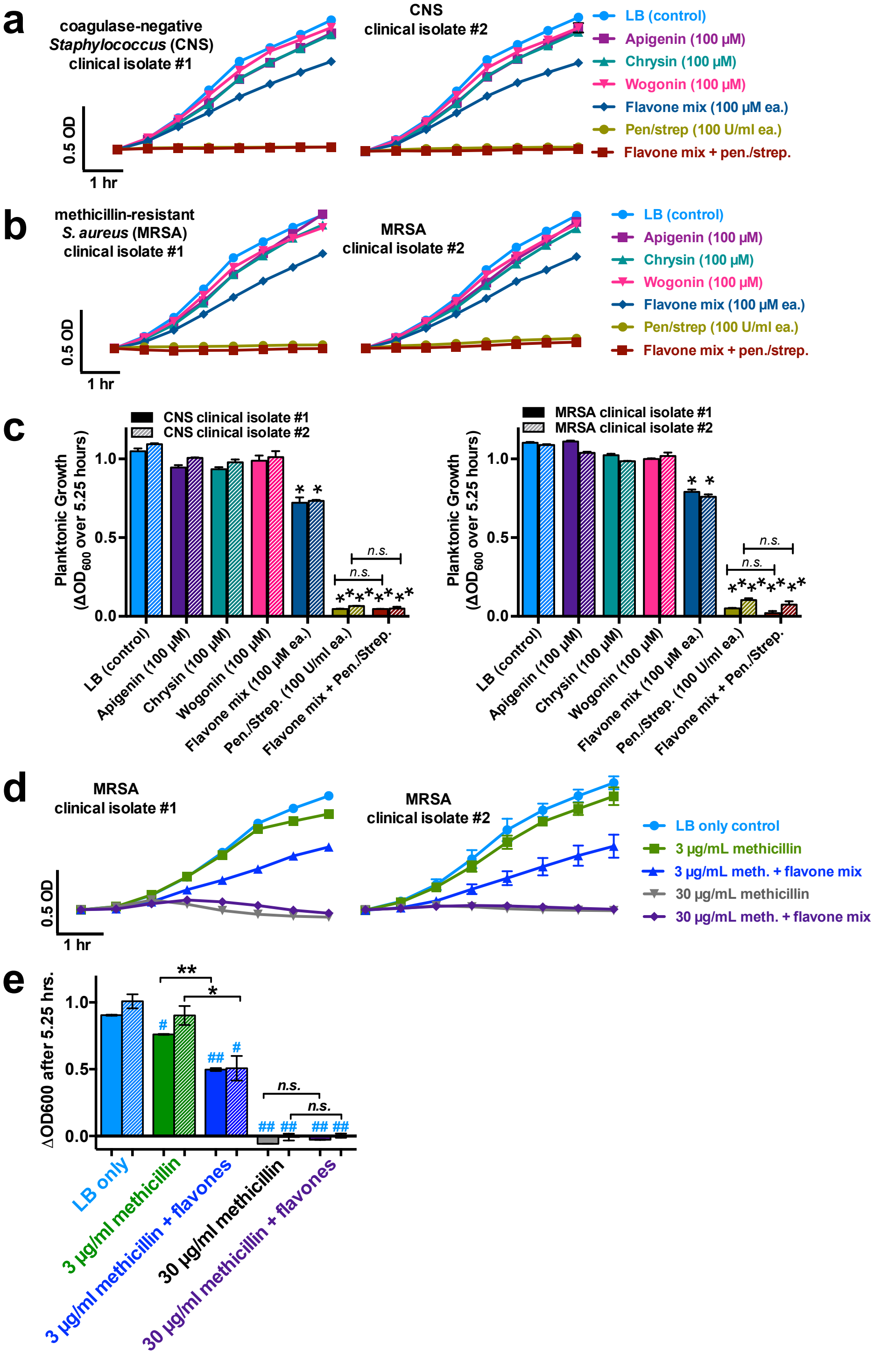

S2 Fig. Effects of flavones on planktonic *Staphylococcus* growth. (A) Planktonic growth curves of two clinical isolates of coagulase-negative *Staphylococcus* (CNS). (B) Planktonic growth curves of two clinical isolates of methicillin-resistant *Staphylococcus aureus* (MRSA). (C) Bar graphs showing planktonic growth over 5.25 hrs. from experiments above (n = 4 experiments for each condition). Individual flavones had no significant effect on planktonic growth, but a mixture of flavones (100 µM each apigenin, chrysin, wogonin) slightly but significantly reduced planktonic growth (p <0.05 vs. control). The changes in OD_{600} after 5.25 hrs. were 1.04 ± 0.02 (CNS #1), 1.09 ± 0.005 (CNS # 2), 1.10 ± 0.005 (MRSA # 1) 1.09 ± 0.004, and 1.09 ± 0.004 (MRSA # 2) in control conditions and 0.72 ± 0.03 (CNS #1; p <0.05 vs control), 0.73 ± 0.01 (CNS # 2; p <0.05 vs control), 0.79 ± 0.015 (MRSA # 1; p <0.05 vs control), and 0.76 ± 0.01 (MRSA # 2; p <0.05 vs control) in cells exposed to apigenin, chrysin, and wogonin (100 µM each). (D) Growth curves of the two MRSA isolates with low (3 µg/ml) and high (30 µg/ml) concentrations of methicillin ± flavones. (E) Bar graphs of data from *D*. Flavones significantly enhanced the inhibition of planktonic growth of both MRSA strains in the presence of 3 µg/ml methicillin (p <0.01 vs. 3 µg/ml methicillin alone for strain #1 [solid bars] and p <0.05 for strain #2 [crossed bars]). The changes in OD_{600} were 0.90 ± 0.004 (MRSA #1) and 0.90 ± 0.05 (MRSA #2) in control conditions, 0.76 ± 0.004 (MRSA #1; p < 0.05 compared with control) and 0.90 ± 0.07 (MRSA #2; *n.s.* compared with control) with 3 µg/ml methicillin, and 0.50 ± 0.01 (MRSA #1; p < 0.05 compared with control; p <0.01 vs methicillin only) and 0.51 ± 0.1 (MRSA #2; p < 0.05 compared with control; p <0.05 vs methicillin only) with flavone mixture plus 3 µg/ml methicillin. For all graphs, * = p <0.05, ** = p <0.01.
