# Supplementary material for: Plant flavones enhance antimicrobial activity of respiratory epithelial cell secretions against Pseudomonas aeruginosa
Source: PLoS One. 2017 Sep 20;12(9):e0185203. doi: 10.1371/journal.pone.0185203 (PMC5607194; doi:10.1371/journal.pone.0185203)
Supplement: S3 Fig — (PDF) [file pone.0185203.s003.pdf]

Supporting Information

Hariri, ***et al.*** **"Plant Flavones Enhance Antimicrobial Activity of Respiratory Epithelial Cell Secretions Against** ***Pseudomonas aeruginosa*"**

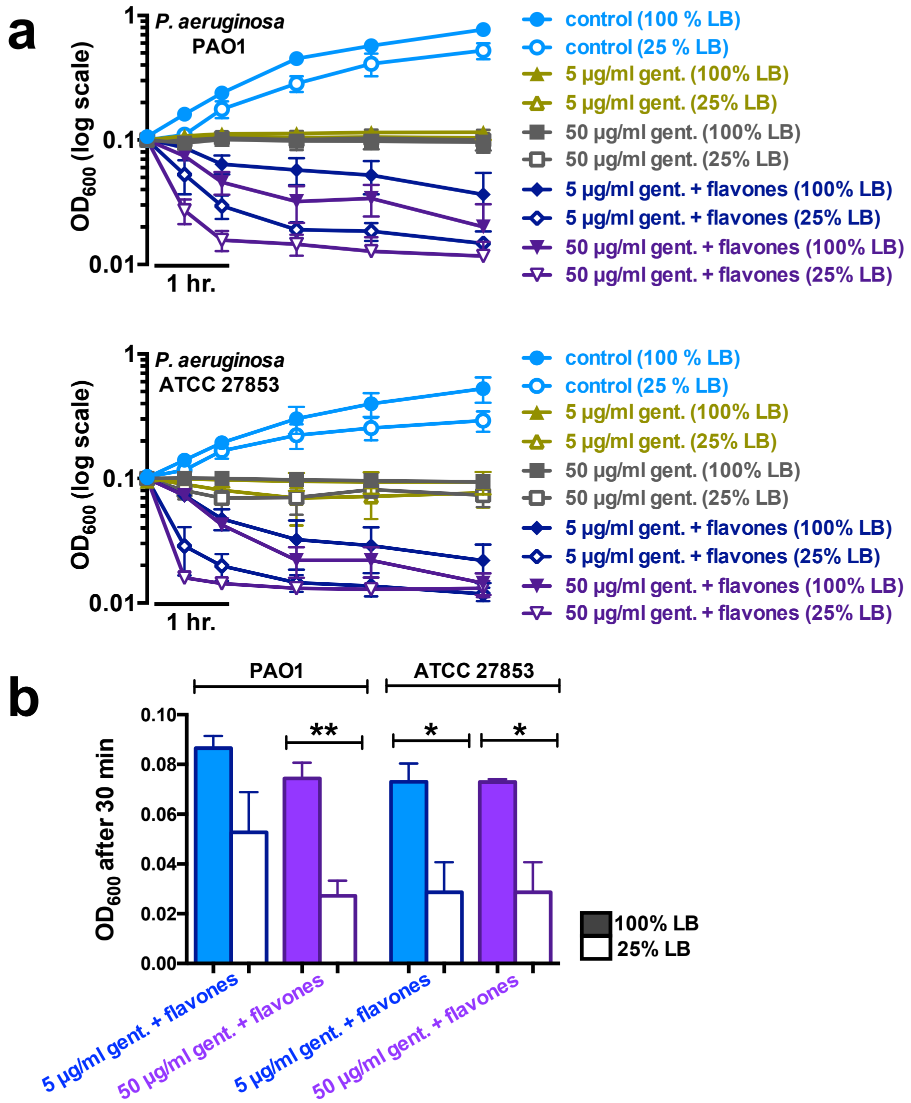

S3 Fig. Effects of ionic strength on flavone + antibiotic cell lysis. (A) Planktonic growth traces of *P. aeruginosa* in the presence of gentamycin or gentamycin + flavone mix in normal (closed symbols) or low (open symbols) ionic strength/osmolality. (B) Bar graphs comparing ΔOD_{600} after 30 min from traces in *C* (starting OD_{600} 0.1) with gentamycin plus flavone. Asterisks denote significance between bracketed groups (* = $p < 0.05$, ** = $p < 0.01$; 1-way ANOVA, Bonferroni posttest), showing dependence of OD_{600} reduction on ionic strength/osmolality.
