# Supplementary material for: Plant flavones enhance antimicrobial activity of respiratory epithelial cell secretions against Pseudomonas aeruginosa
Source: PLoS One. 2017 Sep 20;12(9):e0185203. doi: 10.1371/journal.pone.0185203 (PMC5607194; doi:10.1371/journal.pone.0185203)
Supplement: S4 Fig — (PDF) [file pone.0185203.s004.pdf]

Supporting Information

Hariri, *et al.* "Plant Flavones Enhance Antimicrobial Activity of Respiratory Epithelial Cell Secretions Against *Pseudomonas aeruginosa*"

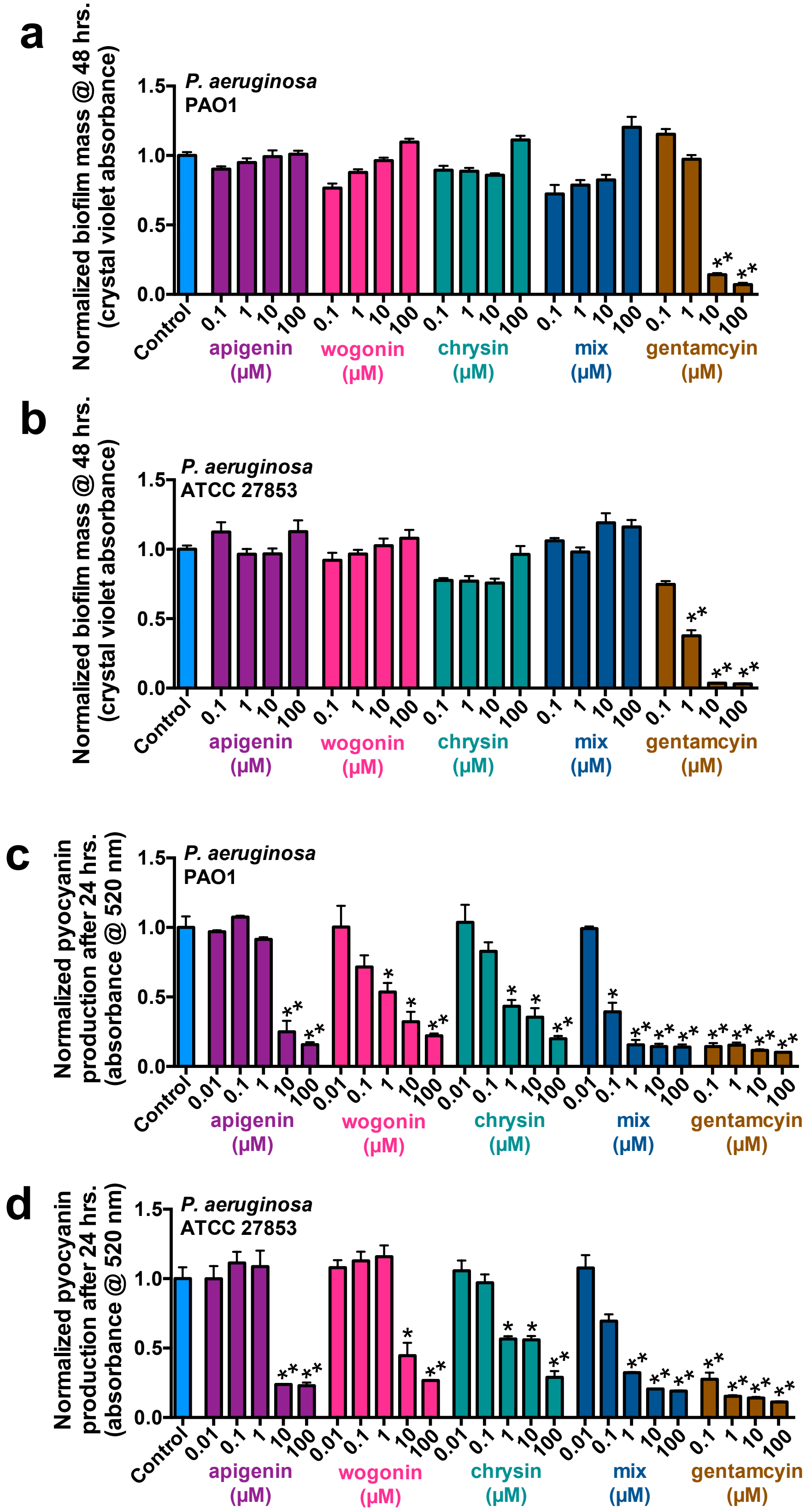

S4 Fig. Bitter flavones do not affect *P.aeruginosa* biofilm formation but do reduce pyocyanin production. (A and B) Results from crystal violet biofilm assays using two strains of P. aeruginosa in the presence of flavones, showing no significant effect of 3 individual flavones or a mix. (C and D), Pyocyanin production was measured from two *P. aeruginosa* strains after 24 hrs. culture in Müeller-Hinton broth. Apigenin, wogonin, and chrysin significantly reduced pyocyanin production at effective concentrations of 1-10 µM. For all graphs, * = $p < 0.05$, ** = $p < 0.01$.
