# Supplementary material for: Plant flavones enhance antimicrobial activity of respiratory epithelial cell secretions against Pseudomonas aeruginosa
Source: PLoS One. 2017 Sep 20;12(9):e0185203. doi: 10.1371/journal.pone.0185203 (PMC5607194; doi:10.1371/journal.pone.0185203)
Supplement: S1 File — (PDF) [file pone.0185203.s005.pdf]

Supporting Information

Hariri, *et al.* "Plant Flavones Enhance Antimicrobial Activity of Respiratory Epithelial Cell Secretions Against *Pseudomonas aeruginosa*"

S1 File. Data values from the main text.

OD_{600} data values from Figure 1: (B) The ΔOD_{600} with PAO1 was 0.60 ± 0.01 (control), 0.62 ± 0.03 (100 µM apigenin; *n.s.* vs. control), 0.51 ± 0.02 (100 µM chrysin; p <0.05 vs. control), 0.42 ± 0.01 (300 µM chrysin; p <0.05 vs. control), 0.43 ± 0.02 (100 µM wogonin; p <0.05 vs. control), 0.44 ± 0.02 (flavone mix; 100 µM each, chrysin, wogonin and apigenin; p <0.05 vs. control), -0.01 ± 0.006 (penicillin + streptomycin; p <0.01 vs. control), -0.15 ± 0.02 (penicillin, streptomycin + flavone mix; p <0.01 vs. control; p < 0.01 vs. pen/strep alone). The change in OD_{600} with ATCC 27853 was 0.36 ± 0.02 (control), 0.33 ± 0.03 (100 µM apigenin; *n.s.* vs. control), 0.28 ± 0.06 (100 µM chrysin; p <0.05 vs. control), 0.06 ± 0.08 (300 µM chrysin; p <0.01 vs. control), 0.15 ± 0.02 (100 µM wogonin; p <0.01 vs control), 0.06 ± 0.02 (flavone mix; p <0.01 vs control), -0.03 ± 0.006 (penicillin + streptomycin; p <0.01 vs control), -0.33 ± 0.02 (penicillin, streptomycin + flavone mix; p <0.01 vs control; p < 0.01 vs pen/strep alone).

OD_{600} data values from Figure 2: (B) Initial rates of OD_{600} decrease (in units/min) over 30 min for PAO1 (n = 3-6 experiments for each condition) were 0.0014 ± 0.0004 (control), 0.0023 ± 0.00096 (chrysin; *n.s.* vs. control), 0.0056 ± 0.001 (apigenin; p <0.05 vs. control), 0.0026 ± 0.00097 (wogonin; *n.s.* vs. control), 0.0044 ± 0.0014 (flavone mix; *n.s.* vs. control), 0.008 ± 0.001 (lysozyme; p <0.05 vs. control), 0.0085 ± 0.00090 (chrysin + lysozyme; p <0.05 vs. control; *n.s.* vs. lysozyme only), 0.0078 ± 0.0012 (apigenin + lysozyme; p <0.05 vs. control; *n.s.* vs. lysozyme only), 0.0062 ± 0.00092 (wogonin + lysozyme; p <0.05 vs. control; *n.s.* vs. lysozyme only), 0.016 ± 0.0037 (flavone mix + lysozyme; p <0.05 vs. control; p <0.05 vs. lysozyme only). Initial rates of OD_{600} decrease for ATCC27853 were 0.00074 ± 0.00043 (control), 0.0019 ± 0.00086 (chrysin *n.s.* vs. control), 0.0028 ± 0.00058 (apigenin *n.s.* vs. control), 0.0017 ± 0.00089 (wogonin; *n.s.* vs. control), 0.0066 ± 0.0013 (flavone mix; p <0.05 vs. control), 0.0073 ± 0.0021 (lysozyme; p <0.05 vs. control), 0.0071 ± 0.00068 (chrysin + lysozyme; p <0.05 vs. control; *n.s.* vs. lysozyme only), 0.0080 ± 0.0014 (apigenin + lysozyme; p <0.05 vs. control; *n.s.* vs. lysozyme only), 0.0084 ± 0.0014 (wogonin + lysozyme; p <0.05 vs. control; *n.s.* vs. lysozyme only), 0.013 ± 0.003 (flavone mix + lysozyme; p <0.05 vs. control; p <0.05 vs. lysozyme only). **(C)** The ΔOD_{600} after 2 hrs. for PAO1 was 0.22 ± 0.04 (control), 0.012 ± 0.05 (chrysin; p <0.05

vs. control), 0.011 ± 0.050 (apigenin; p <0.05 vs. control), 0.037 ± 0.07 (wogonin; *n.s.* vs. control), -0.12 ± 0.08 (flavone mix; p <0.01 vs. control), -0.22 ± 0.02 (lysozyme; p <0.01 vs. control; *n.s.* vs. flavone mix alone), -0.28 ± 0.03 (chrysin + lysozyme; p <0.01 vs. control; *n.s.* vs. lysozyme only), -0.26 ± 0.02 (apigenin + lysozyme; p <0.01 vs. control; *n.s.* vs. lysozyme only), -0.26 ± 0.02 (wogonin + lysozyme; p <0.01 vs. control; *n.s.* vs. lysozyme only), -0.41 ± 0.03 (flavone mix + lysozyme; p <0.01 vs. control; p <0.05 vs. lysozyme only). The ΔOD_{600} after 2 hrs. for ATCC 27853 was 0.17 ± 0.006 (control), -0.07 ± 0.03 (chrysin; p <0.01 vs. control), -0.08 ± 0.01 (apigenin; p <0.01 vs. control), -0.09 ± 0.04 (wogonin; p <0.01 vs. control), -0.18 ± 0.04 (flavone mix; p <0.01 vs. control), -0.23 ± 0.05 (lysozyme; p <0.01 vs. control; *n.s.* vs flavone mix alone), -0.28 ± 0.03 (chrysin + lysozyme; p <0.01 vs. control; *n.s.* vs. lysozyme only), -0.26 ± 0.03 (apigenin + lysozyme; p <0.01 vs. control; *n.s.* vs. lysozyme only), -0.30 ± 0.2 (wogonin + lysozyme; p <0.01 vs. control; *n.s.* vs. lysozyme only), -0.42 ± 0.02 (flavone mix + lysozyme; p <0.01 vs. control; p <0.01 vs. lysozyme only).

Fluorescence values from Figure 3: Relative fluorescence values from GFP in supernatant (n = 6-11 experiments per condition) were 1 ± 0.07 (control), 11.64 ± 1.7 (lysozyme alone), 27.53 ± 2.7 (lysozyme + flavone mix), 5.2 ± 0.6 (flavone mix alone), 1.7 ± 0.1 (apigenin alone), 1.5 ± 0.09 (chrysin alone), 16.6 ± 2.6 (lysozyme + apigenin), 13.7 ± 2.4 (lysozyme + chrysin).

Fluorescence values from Figure 4: Raw NPN fluorescence values for PAO1 were (n = 4-10 experiments per condition) were 5889 ± 124 (control), 20301 ± 413 (lysozyme), 29281 ± 1217 (lysozyme + flavone mix), 21929 ± 1158 (lysozyme + apigenin), 22347 ± 1314 (lysozyme + chrysin), 22347 ± 595 (lysozyme + wogonin), 6111 ± 247 (apigenin only), 5988 ± 146 (chrysin only), 13222 (flavone mix only). Raw fluorescence values for ATCC 27853 (n = 4-10 experiments per condition) were 7360 ± 96 (control), 22725 ± 760 (lysozyme), 31673 ± 689 (lysozyme + flavone mix), 23713 ± 1183 (lysozyme + apigenin), 24309 ± 1475 (lysozyme + chrysin), 25353 ± 2486 (lysozyme + wogonin), 7469 ± 179 (apigenin only), 7406 ± 122 (chrysin only), 15347 (flavone mix only). (B)

CFU values from Figure 5: (A) CFUs recovered (n = 5-7 experiments for each condition) were 19857 ± 1280 (PBS), 14400 ± 2315 (PBS + flavones; *n.s.* vs. PBS alone), 4 ± 2 (50 µg/ml gentamycin; p <0.01 vs control), 1 ± 1 (50 µg/ml gentamycin + flavones; p <0.01 vs control; n.s. vs gentamycin

alone), 1 ± 1 (50% Calu-3 ASL; *p* <0.01 vs control), 1 ± 1 (50% Calu-3 ASL + flavones; *p* <0.01 vs control; *n.s.* vs 50% ASL alone), 3 ± 2 (25% Calu-3 ASL; *p* <0.01 vs control), 2 ± 1 (25% Calu-3 ASL + flavones; *p* <0.01 vs control; *n.s.* vs 25% ASL alone), 2030 ± 998 (12.5% Calu-3 ASL; *p* <0.05 vs. control), 42 ± 20 (12.5% Calu-3 ASL + flavones; *p* <0.05 vs 12.5% ASL alone; *p* <0.01 vs control), 18400 ± 2337 (6.25% Calu-3 ASL; *n.s.* vs. control), 2800 ± 1362 (6.25% Calu-3 ASL + flavones; *p* <0.05 vs control; *p* <0.01 vs 6.25% ASL alone). **(B)** CFUs recovered (n = 4 experiments for each condition) were 23250 ± 4029 (PBS), 17250 ± 2562 (PBS + flavones; *n.s* vs. PBS alone), 3 ± 3 (50% ASL), 5 ± 4 (50% ASL + flavones; *n.s.* vs. 50% ASL alone), 4050 ± 2244 (25% ASL), 88 ± 59 (25% ASL + flavones; *p* <0.01 vs. 25% ASL alone), 26000 ± 3808 (12.5% ASL), and 15500 ± 4173 (12.5% ASL + flavones; *p* <0.05 vs. 12.5% ASL alone).
